# Supplementary material for: Essential newborn care practices and associated factors among home delivered mothers in Guto Gida District, East Wollega zone
Source: PLOS Glob Public Health. 2023 Jan 20;3(1):e0001469. doi: 10.1371/journal.pgph.0001469 (PMC10021559; doi:10.1371/journal.pgph.0001469)
Supplement: S1 Questionnaire — (DOCX) [file pgph.0001469.s001.docx]

**ANNEX I: ENGLISH VERSION QUESTIONNAIRE**

WOLLEGA UNIVERSITY INSTITUTE OF HEALTH SCIENCES DEPARTMENT OF PUBLIC HEALTH

Semi structured English version questionnaire and consent form

Information Sheet

**INTRODUCTION**

**Greeting!** My name is-----------------I am working as data collector for the study being conducted in this kebele by **Mr. Mulugeta Abebe** who is studying for his master’s degree at Wollega university institute of health sciences department of public health postgraduate study. I kindly request you to lend me your attention to explain you about the study and how you have been selected as study participant.

**Study title–** Essential newborn care practice and associated factors among home delivered mothers in Guto Gida district, Nekemte town

**Purpose**- To assess prevalence of essential newborn care practice and its associated factors among home delivered mothers in Guto Gida district, east Wollega zone, Oromia, Ethiopia, 2020.

**Procedure and duration:** First of all we selected you to take part in this study randomly. There are 50 questions to answer, by interviewing you, the questioner will be filled. The interview will take around 30 - 35 minutes.

**Risks:** The risks of being participating in this study are very minimal, only taking your few minutes.

**Benefit:** At this moment you may not get any direct benefit by being involved in this study but the information you provide is very important to solve problems on new-born care practice issue.

**Confidentiality:** The information that you provide us will be confidential. The questioner will be coded to exclude showing your name on questioner and consent form.

**Rights:** Participation in this study is fully voluntary. You have the right to declare not to participate in this study and you have the right to with draw from participating at any time

**Contact address:** If there is any questions or unclear idea any time about the study or the procedures, do not hesitate to contact and speak to Principal investigator, with tell phone number: **0909711313 or e-mail address muller655@gmail.com.**

**Consent form**

I have read this form or it has been read to me in the language I comprehend and understand all condition stated above.

**Are you willing to participate in this study?**

a. **Yes** (continue to interview) b. **No** (stop)

Signature of data collector __________________ Date _________________

Name of **kebele** ______________

| Part 1: Socio Demographic Characteristics of Respondent | | | | | | | | | |
| --- | --- | --- | --- | --- | --- | --- | --- | --- | --- |
| Q | Question | | Answers and codes | | | Go to /Remark | | | |
| 101 | How old are you? | | ___________ In Years | | |  | | | |
| 102 | Religion | | 1. Protestant 2. Orthodox 3. Muslim 4. Wakefata 5. Others | | |  | | | |
| 103 | Ethnicity | | 1. Oromo 2. Amhara 3. Gurage 4. Tigre 5. Others | | |  | | | |
| 104 | What is your marital status? | | 1. Never married 2. Married 3. Divorced 4. Widowed 5. Separated 6. Others(Specify)____________ | | |  | | | |
| 105 | What is your occupation? | | 1. House wife  2. Merchant  3. Government employee  4. Self-Employee  5. Student  6. Others(specify)_____________ | | |  | | | |
| 106 | What is your educational level | | 1. No formal education  2. Primary (1-8)  3. Secondary (9-12)  4. College and above | | |  | | | |
| 107 | How much is your family total monthly income? in ETB | | _________________ | | |  | | | |
| Part 2: Maternal and Neonatal information | | | | | | | | | |
| 201 | What was your age at first delivery? | | ………in year | | |  | | | |
| 202 | How many children do you have (alive)? | | ………. | | |  | | | |
| 203 | Do you have history of neonatal death? | | 1. Yes 2. No | | | If 2 skip 301 | | | |
| 204 | When did the death had occurred | | 1. First day 2. First week 3. Second week  4. Third week 5. Fourth week | | |  | | | |
| 205 | Do you explain the cause of death? | | ………………….. | | |  | | | |
| **Part 3: Reasons of mothers for their home delivery** | | | | | | | | | |
| 301 | | Have you reason for your home delivery? | | | 1. Yes 2. No | If 2 skip 401 | | | |
|  |  | 1. No nearby health facility  2. No transportation  3. Precipitated labor  4. Lack of money  5. No partner for help  6. I don’t want to go health facility  7. Other specify _____________ | | | 1 2  1 2  1 2  1 2  1 2  1 2  1 2 |  | | | |
| Part 4: Assessing mothers status of health service utilization | | | | | | | | | |
| 401 | | Did you have a home visit by HEW and get advice during your current pregnancy and PNC? | | | 1. Yes 2. No | | If 2 skip to 402 | | |
|  |  | 1. Hand washing with soap and water before handling the neonate. | | | 1 2 | |  | | |
|  |  | 2. Keeping the neonate immediately dry and wrapping before delivery of placenta. | | | 1 2 | |  |  |  |
|  |  | 3. Breastfeeding immediately after birth within an hour? | | | 1 2 | |  |  |  |
|  |  | 4.Danger sign of the neonate that need immediate health care | | | 1 2 | |  |  |  |
| 402 | | Did you see anyone for ANC during last pregnancy? | | | 1. Yes 2. No | | | | If 2 skip to 404 |
|  |  | a) Doctor | | | 1 2 | | | |  |
|  |  | b) Midwife/Nurse/HO | | | 1 2 | | | |  |
|  |  | c) HEW | | | 1 2 | | | |  |
|  |  | d)other specify | | | …………… | | | |  |
| 403 | | Where did you receive antenatal care for this pregnancy? | | | 1. Yes 2. No | | | |  |
|  |  | a) Hospital | | | 1 2 | | | |  |
|  |  | b) Health centre | | | 1 2 | | | |  |
|  |  | c) Health post | | | 1 2 | | | |  |
|  |  | d)other specify | | | ………………. | | | |  |
| 404 | | How many weeks pregnant, when you received your first ANC? | | | 1.________weeks  2. Can’t Remember | | | |  |
| 405 | | How many times did you receive antenatal care for this pregnancy? | | | 1. Number of visit___  2. Can’t Remember | | | |  |
| 406 | | Was/were your birth attendant/s had washed their hands and used soap? | | | 1. Yes 2. No | | |  | |
| 407 | | Was/were your birth attendant/s had worn gloves? | | | 1. Yes 2. No | | |  | |
| 408 | | Who attend your current delivery? | | 1. HEW  2. TBA  3. Mother/Mother-in-law  4. Sister/sister-in-law  5. Other female relative  6. No one | | | |  | |
| 409 | | Did you go to health institution for Postnatal check-up? | | | 1. Yes 2. No | | | If 2 skip to Q501 | |
|  |  | 1. How many PNC visit you attend? | | | _____ in number | | |  | |
|  |  | 1. At what hours/day you attend your first PNC check-up after delivery? | | | _____ in hour/ days | | |  |  |

| **Part 5: Assessing the prevalence of breast feeding practice.** | | | |
| --- | --- | --- | --- |
| Q | Question | Response and code | Go to /Remark |
| 501 | Did you give the first breast milk (colostrums) for your baby? | 1. Yes 2. No 3. Can’t Remember |  |
| 502 | When did you give the first breast milk for your baby after delivery? | 1. Within an hour after birth  2. B/n 1hour and 24 hours  3. After 24 hours  4. I didn’t give breast  5. Can’t Remember |  |
| 503 | Did you give additional food for your current baby | 1. Yes 2. No 3. Can’t Remember | If 2/3 skip Q 601 |
|  | a) What additional food did you give for your current baby after birth? | 1. Water 2. Honey 3. Cow milk 4. Infant formula 5. Butter 6. Others(specify) |  |

| **Part 6: Assessing the prevalence of cord care practice** | | | | |
| --- | --- | --- | --- | --- |
| Q | Question | Response and code | | Go to /Remark |
| 601 | What was used to cut the cord? | 1. New razor blade  2. Old Razor blade  3. Can’t Remember  4. Other specify ………… | | If 1/3 skip Q 602 |
|  | a).Was the instrument used to cut cord boiled prior to use? | 1. Yes 2. No 3. Can’t Remember | |  |
| 602 | What was used to tie the cord? | 1. New string or thread  2. Old String, or thread  3. Cord was not tied  4. Can’t Remember  5. Other (specify) __________ | If 1/3/4 skip Q 603 | |
|  | a) Was the tie/string used to tie the cord Washed prior to use? | 1. Yes 2. No 3. Can’t Remember |  | |
| 603 | Did you apply anything on the cord of your baby? | 1. Yes 2. No 3. Can’t Remember | If, 2/3 skip Q 701 | |
|  | a) What did you apply? | 1. Butter  2. Vaseline  3. Ointment/oil  4. Others( specify)________ |  | |

| **Part 7: Assessing the prevalence of the thermal care practice of the neonate** | | | | | |
| --- | --- | --- | --- | --- | --- |
| Q | Question | Response and code | | Go to /Remark | |
| 701 | When did you give bath for the baby after birth? | 1. Within 1 hour  2. B/n 1hour and 24 hours  3. After 24 hour  4. Can’t Remember | |  | |
| 702 | Was the newborn wiped (dried), before the placenta was delivered? | 1. Yes 2. No 3. Can’t Remember | |  | |
| **Part 8: Assessing knowledge and attitude of mothers on ENCP & neonatal danger signs** | | | | | |
| Q | Question | | Response and code | | Go to /Remark |
| 801 | Do you know about care of the mother to her newborn baby? | | 1. Yes 2. No | | If 2 skip to Q 803 |
|  | a) Do you know instruments used during cord cut? | | 1. New razor blade  2. Old Razor blade  3. Don’t know  4. Other specify ………… | |  |
|  | b) Do you know materials used during cord tie? | | 1. New string or thread  2. Old String or thread  3. Cord was not tied  4. Don’t know  5. Other (specify) ____ | |  |
|  | c) Do you know substances should be applied to the cord immediately after cut up to 7 days except ordered medication? | | 1. Nothing  2. Butter applied  3. Vaseline/ointment/oil  4. Don’t Know  5. Other (specify)____ | |  |
|  | d) Do you know how long after birth should the newborn be washed / bathed for the first time? | | 1. Within one hour  2. B/n 1hour and 24 hours  3. After 24 hours  4. Don’t know | |  |
|  | e) Do you know how long after birth the newborn should be breast fed? | | 1. Within one hour  2. B/n 1hour and 24 hours  3. After 24 hours  4. Don’t know | |  |
|  | f) Do you know what new born feed first? | | 1. Sugar water  2. Fresh butter  3. Breast milk /colostrums  4. Plain water  5. Milk (excluding breast milk) 6. Other (Specify)____  7. Don’t know | |  |
| 802 | Is the mother respond correctly above 50% of knowledge related questions? | | 1. Yes 2. No | | If 2 skip to Q 803 |
|  | a) Do you feel they are good intervention to reduce neonatal morbidity and mortality? | | 1. Yes 2. No | |  |
| 803 | Do you know about neonatal danger sign? | | 1. Yes 2. No | | If 2 End of the interview. |
|  | a) Poor sucking or not able feed breast | | 1 2 | |  |
|  | b) Fast breathing | | 1 2 | |  |
|  | c) Hypothermia | | 1 2 | |  |
|  | d) Fever | | 1 2 | |  |
|  | e) Drowsy or unconscious | | 1 2 | |  |
|  | f) Cord bleeding and infection | | 1 2 | |  |
| 804 | If the mother mention 4 or more of any neonatal danger sign? | | 1. Yes 2. No | | If 2 End of the interview. |
|  | a) Do you feel, all of them need urgent medical care”? | | 1. Yes 2. No | |  |

-------------------------------------------------------//------------------------------------------------------------

-----------------------------------------------------//--------------------------------------------------------------

Thank you!!

**ANNEX II: AFAN OROMO VERSION QUESTIONNARE**

Gaafilee odeeffannoo waa’ee Gochaa kunuunsa daa’imman dhalattuuf haadholiin godhanii fi waantoota dhiibbaa geessisan irratti odeeffannoo sassaabuuf qophaa’e

Waraqaa odeeffannoo

**Seensa:** Akkam jirtu? ani maqaan koo Obboo/Aaddee___________ kanan jedhamu odeeffannoo qorannoo waa’ee gocha kunuunsa daa’imman dhalattuuf haadholiin godhanii fi waantoota dhiibbaa geessisan irratti Obboo Mulugeeta Abbabee Yuunibarsiitii Wallaggaa muummee barnoota fayyaa hawaasa irratti gaggeeffamaa jiru irratti odeeffannoo isin irraa funaanuufi.

**Kaayyoo qorannichaa** –kaayyoon qorannnoo kanaa gochaa kunuunsa daa’imman dhalattuuf haadholiin godhanii fi waantoota dhiibbaa geessisan qorachuudha.

**Faayidaa**:Rakkoolee daa’imman dhalattuu mudachuu danda’an ittisuuf gargaara,

**miidhaa:**yeroo kee muraasa kan daqiiqaa 30-35 hin caalleedha fudhachuu.

**Iccittii** – hundinu eegamaadha, akkasumas waraqaa gaaffii irrattis ta’ee unka walii galtee irratti maqaan kee hin katabamu.s

**Mirga** – Qorannicha irratti hirmaachuu, dhiisuu fi addaan kutuu danda’uu kee guutummaatti eegamaadha.

**Qunnamtii** – Adeemsa fi waa’ee qorannichaa irratti gaaffii yoo qabaattan abbaa qorannoo kanaa kan ta’e Obboo Mulugeeta Abbabee karaa lakkofsa bilbilaa: +251909711313 yookiin e-Mail muller 655@gmail.com Kanaan argachuu ni dandeessuu.

**Unka Walii galtee**

Wantoota armaan olitti ibsamaniijiran hunduu siif hubatameejiraa? Qarannoo kana irratti hirmaachuuf fedhii qabdaa?

a) **Eeyyee** (itti fufi) b) **Lakki**( irra darbi)

Mallattoo nama odeeffannoo funaanee________________ guyyaa_______________

Maqaa gandaa ______________________

| **Kutaa I: Odeeffannoo haala hawaasummaa fi dinagdee ilaalchisee.** | | | | | | | | | | | | | | | | | | | | | | | | | | | | | |
| --- | --- | --- | --- | --- | --- | --- | --- | --- | --- | --- | --- | --- | --- | --- | --- | --- | --- | --- | --- | --- | --- | --- | --- | --- | --- | --- | --- | --- | --- |
| Q | | | Gaaffilee | | | | Deebii fi koodii | | | | | | | | | Itti darbi | | | | | | | | | | | | | |
| 101 | | | Umriin | | | | ___________ waggaadhan | | | | | | | | |  | | | | | | | | | | | | | |
| 102 | | | Amantaa | | | | 1. Pirotestaantii  2. Ortodoksii  3. Musliima  4. Waaqeffata  5. Kan biroo(ibsi)___________ | | | | | | | | |  | | | | | | | | | | | | | |
| 103 | | | Sabummaa | | | | 1. Oromoo  2. Amahara  3. Guraage  4. Tigree  5. Kan biroo(ibsi)_______ | | | | | | | | |  | | | | | | | | | | | | | |
| 104 | | | Haala fudhaa fi heerummaa ilaalchisee | | | | 1. Hin heerumne  2. Heerume  3. Wal hiikne  4. Narraa du’e  5. Walbirra hinjjiru | | | | | | | | |  | | | | | | | | | | | | | |
| 105 | | | Sadarkaa barnootaa | | | | 1. Barnoota idilee kan hin qabne  2. Barnoota sadarkaa 1ffaa  3. Barnoota sadarkaa 2ffaa  4. Dippiloomaa fi isaa ol ________ | | | | | | | | |  | | | | | | | | | | | | | |
| 106 | | | Hojiin kee maali | | | | 1. Haadha manaa  2. Daldaalttuu  3. Hojjattuu mootummaa  4. Hojii dhuunfaa  5. Barattuu  6. Kan biroo(ibsi)___________ | | | | | | | | |  | | | | | | | | | | | | | |
| 107 | | | Galiin dimshaashaa maatii kee ji’aatti meeqa? | | | | ----------------- Maallaqa Ityopiatin | | | | | | | | |  | | | | | | | | | | | | | |
| **Kutaa II: Odeeffannoo Hadholi fiDa’imaani** | | | | | | | | | | | | | | | | | | | | | | | | | | | | | |
| 201 | Yeroo da’uumsa kee isa jalqabaatti umriin kee meeqa ture? | | | | | | | | | | | Waggaa ------------- | | | | | | | | | | |  | | | | | | |
| 202 | Ijoolee meeqa qabdaa? (kan lubbuun jiran) | | | | | | | | | …………… | | | | | | | | | | | | |  | | | | | | |
| 203 | kanaan dura da’imni lubbuun dhalatee/dhalattee si harkaa bahe jiraa?) | | | | | | | | | 1. Eeyyee 2. Lakkii | | | | | | | | | Yoo 2 Q301 tti darbi | | | | | | | | | | |
| 204 | Gaaffiinkee lakk. 203 irratti deebiinkee“eeyyee “yoo ta’e, umuriin daa’imaa meeqa turee? | | | | | | | | | 1. Guyyaa jalqabaa 2.Torban jalqabaa  3. Turban lamatti 4.Torban sadiitti  5. Turban afuritti | | | | | | | | | | | | | |  | | | | | |
| 205 | Sababni daa’imnikee si harkaa bahef/du’eef maali? | | | | | | | | | Ibsi……………. | | | | | | | | | | | | | |  | | | | | |
| **Part III: Sababa Manatti Da’auu** | | | | | | | | | | | | | | | | | | | | | | | | | | | | | |
| 301 | Manatti da’auuketif sababa qabda? | | | | | | | | | | | | 1. Eeyyee 2. Lakkii | | | | | | Yoo 2 Q401 tti darbi | | | | | | | | | | |
|  | a) Eyyee,yoot a’ee, Manni yaalaa nutti dhiyoon hin jiru | | | | | | | | | | | | 1 2 | | | | | |  | | | | | | | | | | |
|  | b) Eyyee,yoot a’ee, Geejibni hin jiru | | | | | | | | | | | | 1 2 | | | | | |  |  |  |  |  |  |  |  |  |  |  |
|  | c) Eyyee,yoot a’ee, Ciniinsuu tasaa ture | | | | | | | | | | | | 1 2 | | | | | |  |  |  |  |  |  |  |  |  |  |  |
|  | d) Eyyee,yoot a’ee, Maallaqni hin jiru | | | | | | | | | | | | 1 2 | | | | | |  |  |  |  |  |  |  |  |  |  |  |
|  | e) Eyyee,yoot a’ee, namni na gargaaru hin jiru | | | | | | | | | | | | 1 2 | | | | | |  |  |  |  |  |  |  |  |  |  |  |
|  | f) Eyyee,yoot a’ee, Mana yaalaa deemuu hin feene | | | | | | | | | | | | 1 2 | | | | | |  |  |  |  |  |  |  |  |  |  |  |
|  | g) Eyyee,yoot a’ee, Kan biroo(ibsi) | | | | | | | | | | | | ………. | | | | | |  |  |  |  |  |  |  |  |  |  |  |
| **Part VI::Odeeffanoo waa’ee Ittfayyadamii’nsa tajaajila fayyaa ilaalchisee** | | | | | | | | | | | | | | | | | | | | | | | | | | | | | |
| 401 | Yeroo ulfaa keetii/ daa’umsaa booda, hojjattootni ekisteenshinii fayyaa mana kee daawwatanii waa’ee kunuunsa daa’immaniif kennamu gorsa siif laatanii beekuu? | | | | | | | | | | | | 1. Eeyyee  2. Lakki | | | | | | Yoo 2 Q402tti darbi | | | | | | | | | | |
|  | 1. Daa’imman qabuun | | | | | | | | | | | | 1 2 | | | | | |  | | | | | | | | | | |
|  | 2. Daa’imman reefu dhalattu osoo hobbatiin hin raawwatamin , dafanii qoorsuu fi huccuu itti uffisuu | | | | | | | | | | | | 1 2 | | | | | |  | | | | | | | | | | |
|  | 3. Daa’imman akkuma dhalataniin yeroo hanga sa’aatii tokkoo keessatti harma hoosisuu jalqabu | | | | | | | | | | | | 1 2 | | | | | |  | | | | | | | | | | |
|  | 4.Mallattoolee hamoo tajaajila fayyaa ariifachiisaa barbaadan | | | | | | | | | | | | 1 2 | | | | | |  | | | | | | | | | | |
| 402 | Yeroo ulfaa keeti tajaajila hordoffii ulfaatiif argatte jirtaa? | | | | | | | | | | | | Eyee =1 Lakki =2 | | | | | Yoo 2 Q404 tti darbi | | | | | | | | | | | |
|  | a) Eyee yoo ta`ee eenyun? Doktora | | | | | | | | | | | | 1 2 | | | | |  | | | | | | | | | | | |
|  | b) Eyee yoo ta`ee eenyun? Midwayifi/Nursi | | | | | | | | | | | | 1 2 | | | | |  |  |  |  |  |  |  |  |  |  |  |  |
|  | c) Eyee yoo ta`ee eenyun? HEW | | | | | | | | | | | | 1 2 | | | | |  |  |  |  |  |  |  |  |  |  |  |  |
|  | d) Eyee yoo ta`ee eenyun? Kan bira (Ibsi) | | | | | | | | | | | | _________ | | | | |  |  |  |  |  |  |  |  |  |  |  |  |
| 403 | Yeroo ulfaa keeti Essaayyu (bakka) deemte tajaajila hordoffii ulfaa argatte jirttaa? bakka kamfaa deemte? | | | | | | | | | | | | 1. Eyee  1. Lakki | | | | |  | | | | | | | | | | | |
|  | a) Eyee yoo ta`ee Hospitaala | | | | | | | | | | | | 1 2 | | | | |  | | | | | | | | | | | |
|  | b) Eyee yoo ta`ee Bufata fayyaa | | | | | | | | | | | | 1 2 | | | | |  |  |  |  |  |  |  |  |  |  |  |  |
|  | c) Eyee yoo ta`ee Kellaa fayyaa | | | | | | | | | | | | 1 2 | | | | |  |  |  |  |  |  |  |  |  |  |  |  |
|  | d). Eyee yoo ta`ee Kan biraa (Ibsi) | | | | | | | | | | | | ______ | | | | |  |  |  |  |  |  |  |  |  |  |  |  |
| 404 | Yeroo jalqaba tajaajila hordoffi fayyaa ulfaa  Argattetti, ulfi kee ji’a meeqa ture? | | | | | | | | | | | | 1.______ __Ji’an  2. Lakki hinbeku | | | | |  | | | | | | | | | | | |
| 405 | Yeroo meeqaaf tajaajila hordoffii fayyaa ulfaa argattee? (kardi isii irraa mirkanessi) | | | | | | | | | | | | 1.Lakk meqa ___  2. Lakki hinbeku | | | | |  | | | | | | | | | | | |
| 406 | Deessiftun (deessiftoonni) kee harka isaanii saamunadhan dhiqatanii? | | | | | | | | | | | | 2. Eyyee  2. Lakki miti | | | | |  | | | | | | | | | | | |
| 407 | Deessiftun (deessiftoonni) kee lastika (glaavi) harkaa godhatanii turanii? | | | | | | | | | | | | 2. Eyyee  2. Lakki miti | | | | |  | | | | | | | | | | | |
| 408 | Daa’imni akka dhalateen eenyutu gargaarsa godhefe? | | | 1. Hojjettuu ekstenshini fayyaa 2. Deessistuu aadaa  3. Hadha/hadha buddena 4. Obboletti  5. Firaa kee (dhalaa ) 6. Nam-tokkolle | | | | | | | | | | | | | |  | | | | | | | | | | | |
| 409 | Hordoffi daa’umsaa boodaaf ogeessan fayyaan ilaalamuuf gara dhaabbata fayyaa deemte? | | | | | | | | | | | | | 1. Eyyee  2. Lakki miti | | | | Yoo 2 Q501 tti darbi | | | | | | | | | | | |
|  | **a) Eyyee,**yoot a’ee Yeroo meeqaffi ? | | | | | | | | | | | | | _____ Lakk | | | |  | | | | | | | | | | | |
|  | **b) Eyyee,**yoot a’ee guyyaa meeqaffaatti ? | | | | | | | | | | | | | _____ guyyaan | | | |  | | | | | | | | | | | |
| **Kutaa V: Gochaa haadholiin da’uumsa booda dafanii harma hoosisuu jalqabuu irratti** | | | | | | | | | | | | | | | | | | | | | | | | | | | | | |
| Q | Gaaffilee | | | | | | | Deebii fi koodii | | | | | | | | | | Itti darbi | | | | | | | | | | | |
| 501 | Aannan harmaa isa jalqabaa(silga) mucaa keef kenniteettaa? | | | | | | | 1. Eeyyee 2. Lakki 3. Hin yaadadhu | | | | | | | | | |  | | | | | | | | | | | |
| 502 | Aannan harmaa jalqabaa mucaa keef yeroo akkamii kenniteef? | | | | | | | 1. Da’uumsa booda sa’aatii 1 keessatti  2. Da’uumsa booda sa’aatii 1fi sa’aatii 24 Giddutt  3. Da’uumsa booda sa’aatii 24’n booda  4. Mucaankoo harmaa hin hodhu  5. Hin yaadadhu | | | | | | | | | | | | | |  | | | | | | | |
| 503 | Da’umsaa booda harmakee malee nyaata biroo mucaa keef kennitteettaa? | | | | | | | 1. Eeyyee 2. Lakki 3. Hin yaadadhu | | | | | | | | | Yoo 2/3 Q601 tti darbi | | | | | | | | | | | | |
|  | **a) Eyyee,**yoot a’ee maali? | | | | | | | 1. Bishaan 2. Damma  3. Aannan sa’aa 4 Foormulaa aannan daa’immanii  5. Dhadhaa 6. Kan biroo(ibsi)______ | | | | | | | | | | | | | | | | | |  | | | |
| **Kutaa VI: Gochaa haadholiin kunuunsa handhuura(cord care) daa’imman dhalattuuf godhan ilaalchisee** | | | | | | | | | | | | | | | | | | | | | | | | | | | | | |
| Q | - Gaaffilee | | | | Deebii fi koodii | | | | | | | | | | | | | | Itti darbi | | | | | | | | | | |
| 601 | Hidda handhura muruuf maal fayyadamtanii turtan? | | | | 1. Haaddu/shigree haaraa 2. Haddu/shigree moofa  3. Hinbeku/hin yaadadhu 4. Kan biraa (Ibsi) ___ | | | | | | | | | | | | | | Yoo 1/3- 602tti darbi | | | | | | | | | | |
|  | a) Meshaan isin hidda handhuuraa muruuf itti fayyadamtan Haddu/shigree moofa yoo ta’ee dursee danfifamee ture? | | | | | | | | | | 1. Eyyee 2. lakkimiti  3. Hin yaadadhu | | | | | | | |  | | | | | | | | | | |
| 602 | Yeroo handhurri muramu hiddi handhura maalin guduunfamee (hidhame) ture? | | | | 1. Jirbi haaraawaa 2. Jirbi moofa  3. Handhurri hin hidhamne 4. Hin yaadadhu  5. Kan biraa (Ibsi) ______ | | | | | | | | | | | | | | Yoo 1/3/4- 603tti darbi | | | | | | | | | | |
|  | a) Kanni hidda handhuuraa guduunfufi ittiin fayyadamtan yoo jirbi moofa ta’ee durse danfifamee ture? | | | | | | | | | | 1. Eyyee 2. Lakkimiti  3. Hin yaadadhu | | | | | | | |  | | | | | | | | | | |
| 603 | Handhuura(cord) mucaa keetii waan irra keesse yookin itti dibde jiraa? | | | | | | | | | | 1. Eeyyee 2. Lakki 3. Hin beeku | | | | | | | | Yoo 2/3 701tti darbi | | | | | | | | | | |
|  | a) **Eyyee,**yoot a’ee maali? Maal irra keesse yookin itti dibde? | | | | 1. Dhadhaa 2. Vaasiliinii  3. Dibata/zayita 4. Kan biroo( ibsi)__ | | | | | | | | | | | | | |  | | | | | | | | | | |
| **Kutaa VII: Gochaa haadholiin ho’iinsa daa’imman reefu dhalatanii eeguf ,kunuunsa (thermal care) godhan ilaalchisee** | | | | | | | | | | | | | | | | | | | | | | | | | | | | | |
| Q | | Gaaffilee | | | | Deebii fi koodii | | | | | | | | | | | | | | | | | | | Itti darbi | | | | |
| 701 | | Da’uumsaa booda yeroo akkami keessatti mucaa kee qaama dhiqxaa? | | | | 1. Sa’aatii tokko keessat 2. Sa’aatii 1fi sa’aatii 24 Giddutti  3. Sa’aatii 24’n booda 4. Hin yaadadhu | | | | | | | | | | | | | | | | | | | | | | |  |
| 702 | | Osoo hobbatiin hin raawwatamin , daa’imni dhalate qoorsamee turee? | | | | 1. Eyyee 2. Lakki miti 3. Hin yaadadhu | | | | | | | | | | | | | |  | | | | | | | | | |
| **Kutaa VIII: Beekumsa haadholiin, kunuunsa daa’imman reefu dhalatanii fi mallattoo hamoo/sodaachisoo daa’imman reefu dhalatanii irratti qaban ilaalchisee.** | | | | | | | | | | | | | | | | | | | | | | | | | | | | | |
| Q | | Gaaffilee | | | | | | | Deebii fi koodii | | | | | | | | | | | Itti darbi | | | | | | | | | |
| 801 | | Waa’ee kunuunsa haadholiin daa’imman reefu dhalataniif kennanii beektaa? | | | | | | | 1. Eeyyee  2. Lakki | | | | | | | | | | | Yoo 2  803tti darbi | | | | | | | | | |
|  |  | a) Eyyee,yoot a’ee, Hidda handhuura daa’ima muruuf maal akka fayyadamuqabnu beektaa? | | | | | | | 1. Haddu/shigree haaraa 2. Haddu/shigree moofa  3. Hinbeku 4. Kan biraa (Ibsi) ___ | | | | | | | | | | | | | | | | | | |  | |
|  |  | b) Eyyee,yoot a’ee, Yeroo handhurri da’aimma muramu hiddi handhura maalin akka hidhamuqabuu beektaa? | | | | | | | 1. Jirbi haaraawaa 2. Jirbi moofa  3. Handhurri hin hidhamne 4. Hinbekku  5. Kan biraa (Ibsi) _______ | | | | | | | | | | | | | | | | | |  | | |
|  |  | c) Eyyee,yoot a’ee, Handhuurri erga muramee wanti itti dibamuu jiraa? | | | | | | | 1. Homtuu hin dibamu 2. Dhadhaa  3. Vaaziliinii /Dibata/zayita 4. Hin beeku  5. Kan biroo (ibsi) | | | | | | | | | | | | | | | | | |  | | |
|  |  | d) Eyyee,yoot a’ee, Daa’imni reefuu dhalate yeroo akkami keessatti dhiqamuu qabu? | | | | | | | 1. Sa’aatii tokko keessatti  2 .Sa’aatii 1fi sa’aatii 24 Giddutti  3. Sa’aatii -24 booda  4. Hin beeku | | | | | | | | | | | | | | | | | |  | | |
|  |  | e) Eyyee,yoot a’ee, Daa’imni reefu dhalate yeroo hammamii keessatti harma hodhuu jalqabuu qaba? | | | | | | | 1. Sa’aatii tokko keessatti  2. Sa’aatii 1fi sa’aatii 24 Giddutti  3. Sa’aatii -24 booda  4. Hin beeku | | | | | | | | | | | | | | | | | |  | | |
|  |  | f) Eyyee,yoot a’ee, Daa’imni reefu dhalatee yeroo jalqabaaf maaltu kennamuufii qaba? | | | | | | | 1. Bishaan sukkaaraa qabu 2. Dhadhaa haaraa  3. Aannan harmaa / silga 4. Bishaan lagaa  5. Aannan ( harma alaa) 6. Kan biroo (ibsi)...  7. Hin beeku | | | | | | | | | | | | | | | | | |  | | |
| 802 | | Yoo haati Daa’ima persenta 50 (shantamaa) oli gaaffii beekumsaa kununsa Daa’ima da’umsaa booda gochaa irratti hundaa’e deebiste? | | | | | | | | | | | | | 1. Eyyee  2. Lakki miti | | | | | | Yoo 2  803tti darbi | | | | | | | | |
|  |  | a) Eyyee,yoot a’ee, Gaaffii kana gaafadhu” Kunuunsi daa’imanii du’aa fi dhukkuba daa’immanii ni hirrisa jettee amantaa? | | | | | | | | | | | | | 1. Eyyee 2. Lakki miti | | | | | | | | | | | |  | | |
| 803 | | Mallattoolee balaafamoo ykn sodaachisoo ykn hamoo Daa’imni reefuu dhalatee beektaa? | | | | | | | | | | | | | 1. Eeyyee  2. Lakki | | | | | | Yoo 2 ta’ee dhumeera | | | | | | | | |
|  |  | a) Eyyee,yoot a’ee, Fedhii harma hodhuu dhabuu yookin hodhuu dadhabuu | | | | | | | | | | | | | 1 2 | | | | | |  | | | | | | | | |
|  |  | b) Eyyee,yoot a’ee, Daddafanii arganuu | | | | | | | | | | | | | 1 2 | | | | | |  |  |  |  |  |  |  |  |  |
|  |  | c) Eyyee,yoot a’ee, Qorriinsa qaamaa | | | | | | | | | | | | | 1 2 | | | | | |  |  |  |  |  |  |  |  |  |
|  |  | d) Eyyee,yoot a’ee, Ho’iinsa qaamaa | | | | | | | | | | | | | 1 2 | | | | | |  |  |  |  |  |  |  |  |  |
|  |  | e) Eyyee,yoot a’ee, Humna dhabuu yookin of wallaaluu (of irraanfachuu) | | | | | | | | | | | | | 1 2 | | | | | |  |  |  |  |  |  |  |  |  |
|  |  | f) Handhuurri dhiiguu fi infeekshinii godhachuu | | | | | | | | | | | | | 1 2 | | | | | |  |  |  |  |  |  |  |  |  |
| 804 | | Mallato balaa ciima daa’ima kessaa haati mucaa 4 ykn isaa oli yootarreessitte,gaaffii kana gafaadhu | | | | | | | | | | | | | 1. Eyyee  2. Lakki miti | | | | | | Yoo 2 ta’ee dhumeera | | | | | | | | |
|  |  | a) Eyyee,yoot a’ee, ‘Mallattoon balaa ciima tarreessite kun hundi isaa yaalii hattattamaa hinbarbaadu jettee amantaa”? | | | | | | | | | | | | | 1. Eyyee 2. Lakki miti | | | | | | | | | | | |  | | |

--------------------------------------------------//------------------------------------------------------ ----------

---------------------------------------//------------------------------------------------------

Galatoomaa!!
