# Supplementary material for: Essential newborn care practices and associated factors among home delivered mothers in Guto Gida District, East Wollega zone
Source: PLOS Glob Public Health. 2023 Jan 20;3(1):e0001469. doi: 10.1371/journal.pgph.0001469 (PMC10021559; doi:10.1371/journal.pgph.0001469)
Supplement: Attachment — Submitted filename: Response to Reviewers.docx [file pgph.0001469.s003.docx]

**Author’s response to reviews**

**Title:** Essential newborn care practices and associated factors among home delivered mothers in Guto Gida District, East Wollega Zone

**Authors:**

Mulugeta Abebe ([mulugetaabebe2012@gmail.com](mailto:mulugetaabebe2012@gmail.com))

Gemechu Kejela ([gemechukejela86@gmail.com](mailto:gemechukejela86@gmail.com))

Melese Chego (melesegershom@gmail.com)

Markos Desalegn ([markosdesalegn@gmail.com)](mailto:markosdesalegn@gmail.com)2)

Version: 1 Date: 19 September 2022

Dear Editor, thank you for providing us an opportunity to revise the paper entitled **" Essential newborn care practices and associated factors among home delivered mothers in Guto Gida District, East Wollega Zone"**. We have revised the paper to address the comments of the reviewers. Below is our point-by-point response to the comments.

Below please find our responses to the editor and three reviewers’ comments.

**Responses to the editor's comments**

Thank you very much for your valuable comments.

1. After incorporating all the comments provided by editor and reviewers, grammatical and typical errors in the manuscript are thoroughly edited.
2. All changes to the manuscript are indicated in the text by highlighting with yellow color.

**Response to the Editor’s comments**

Thank you very much for your valuable comments.

1. **Comment-1:** Abstract – In the following lines of results section, “Women who were advised on essential newborn care practice during a home visit by a health extension worker (3.45 p 0.01), women who attended antenatal care during their current pregnancy (1.79 p 0.01), and women who were attended at their birth by a health extension worker (3.29 p 0.01) were more likely to practice essential newborn care.”, the authors must provide the 95% CI for the association estimates and avoid p-values.

**Response to comment 1:** Thank you very much for your valuable comment. 95% CI for the association estimates is provided, and p-values are deleted.

2. **Comment 2:** Methods section of the main manuscript – The authors write, “Women in the reproductive age group (15–49), who resided in the study area for at least six months, were reported to have delivered an alive baby within six months of postpartum and might have had an infant loss prior to the study period. mothers who were seriously ill, had a history of mental illness, and were unable to care for their infants, or care givers/guardians who cared for infants”. It seems that they are trying to describe the inclusion and exclusion criteria but these sentences aren’t conveying the information. Please rewrite.

**Response to comment 2:** Thank you for your valuable comment. The idea was about inclusion and exclusion criteria. In the revised version, the paragraph is modified in the main manuscript.

3. **Comment 3:** Sample size calculation has been done to estimate the prevalence of the adoption of essential newborn care practices and not for the association analysis that they perform subsequently. This potentially could lead to lack of adequate power and missing out on some other risk factors with small effect sizes. This should be mentioned in the limitation section.

**Response to comment 3:** thank you for your comment. The issue is included in to the limitation section

4. **Comment 4:** The numbering of sections and subsections may be removed.

**Response to comment 4:** Thank you for the comment. The numbering of sections and subsections are removed from the revised version of the manuscript.

5. **Comment 5:** The lists of dependent and independent variables may either be shifted to a table or written in to a narrative form, rather than bulleted lists.

**Response to comment 5:** thank you very much. It is corrected according to the given comment.

6. **Comment 6:** Abbreviations need not be listed, better be avoided and if used, should be expanded at the first instance of usage.

**Response to comment 6:** Thank you for your valuable comment. It is corrected accordingly.

7. **Comment 7:** In table-8, it is better to avoid p-values, if the author wants to present, then please give the actual p-value instead of mentioning <0.01 etc.,

**Response to comment 7:** Thank you very much. The p-value is avoided from Table 8.

**Response to Reviewer 1’s comments**

Dear Reviewer, thank you very much for your valuable and constructive comments that can improve the quality of this paper. The response to the comments are given as follows.

**Abstract**

i. **Method section**
1. **Comment 1:** In the method section, no need to write about software details.

**Response to comment 1:** Thank you very much for your comment. The software detail is removed from the method part.

2. **Comment 2:** Focus on description of the protocol

**Response to comment 2:** the description of the protocol is modified.

ii. **Result section**
1. **Comment 1:** In the results section, expression values can be changed.
a. 95% CI instead of CI

**Response:** CI is replaced by 95% CI in the revised version of the main manuscript

b. Write [ a vs.b ; p:0.01] format

**Response:** The format is corrected based on the given comment.

iii. **Conclusion section**
a. **Comment:** The world health organization should be written as World Health Organization

**Response:** Thank you. It is corrected based on the given comment.

b. **Comment:** Recommendations can’t be in conclusion.

**Response:** Recommendation is removed from the conclusion section.

c. **Comment:** Only findings of the study need to be described

**Response:** Only findings are described in the conclusion section of the revised manuscript.

**Introduction**

1. **Comment 1:** Introduction section looks a little lengthy

**Response to comment 1:** Thank you. The introduction section is shortened in the revised manuscript.

2. **Comment 2:** The introduction section should focus on the basis for the study; shouldn’t comment about

**Response to comment 2:** Thank you very much. The introduction section is modified based on the given comment.

3. **Comment 3:** Introduction part 2nd line: “the care they receive from delivery up to 28 days postpartum”
   Need to change it. We are focusing on the neonatal care. Authors can write it as till 28 days of life. Postpartum period refers to maternal care

**Response to comment 3:** Thank you! The sentence is corrected based on the given comment.

**Methodology**

1. **Comment 1:** A little detail about existing care available for the population can be mentioned and also, Existing health care personnel manpower details can be mentioned

**Response to comment 1:** Thank you very much for your comment. We included the health institutions found in the district and some of the health personnel. However, since the study is community based, detail description of the health facilities and health personnel may have no much value. This is way detailed description was not given.

2. **Comment 2:** WHO includes following in essential newborn care:

- Immediate care at birth (delayed cord clamping, thorough drying, assessment of breathing, skin-to-skin contact, early initiation of breastfeeding)
- Thermal care
- Resuscitation when needed
- Support for breast milk feeding
- Nurturing care
- Infection prevention
- Assessment of health problems
- Recognition and response to danger signs
- Timely and safe referral when needed

Authors need to mention the reason for choosing limited items

**Response to comment 2:** Thank you for your comment. As indicated, WHO uses different items to measure essential newborn care practice. However, all the rest items are more or less included under the three items used in the current study and they are considered as the core practices. Most references used in the current study also used the same item (three items).

**Results**

1. **Comment 1:** Expression of variable needs to be changed; should be according to the author’s guidelines.

**Response to comment 1:** Thank you. The expression of variables is changed based on the given comment.

2. **Comment 2:** Husband or spouse education details might help; Also highest educational status in the family will also help in understanding the mother’s family status. Also we can utilize their knowledge to look the ENC practices; If author’s have the details, they can mention.

**Response to comment 2:** Thank you for your valuable comment. Frankly speaking, the data is not collected on the mentioned variable, even if they are very important to indicate ENC practice.

**Discussion**

1. **Comment 1:** Authors should try to describe and compare the results with other studies. No description of other study details and values. Also authors should tell how the present study fares against them in terms of strengths and limitations.

**Response to comment 1:** Thank you for your valuable comments. The discussion section is modified based on the given comments

**Limitations**

1. **Comment 1:** The design of the study has inherent limitations; Authors need to mention it
   o Recall bias also need to be mentioned

**Response to comment 1:** Thank you for your comment. The limitation section is modified based on the given comments.

**General comment**

Typo errors and grammar needs to be corrected; 3 level heading policy doesn't require authors to have the numbering; It can be removed.

Authors can write a footnote below each table: need to expand abbreviations used in the table

**Response to the general comment:** Thank you for your constructive comments. The grammatical errors and typos throughout the manuscript are corrected. Numbering is removed from the heading and subheadings. Also, Abbreviations used in the table are expanded as a footnote under the table.

**Once again, thank you very much for your constructive comments!**

**Response to Reviewer 2’s comments**

Dear Reviewer, thank you very much for your valuable and constructive comments that can improve the quality of this paper. The response to the comments are given as follows.

**Introduction**

1. **Comment 1:** The introduction explains the importance of Essential newborn care. The justification for the study is written in a lengthy way which could be shortened.

**Response to comment 1:** Thank you again for your comment. The justification for the study is shortened.

**Methods and Materials**

1. **Comment 1:** The primary and secondary objectives of the study were not explicitly mentioned.

**Response to comment 1:** The primary and secondary objectives of the study are explicitly mentioned at the end of the introduction section.

2. **Comment 2:** Inclusion and exclusion criteria were not mentioned clearly.

**Response to comment 2:** Thank you for your comment. The inclusion and exclusion criteria are modified based on the given comment.

3. **Comment 3:** Sampling methods and study design were explained in detail.

**Response to comment 3:** Thank you. Sampling methods and study design are modified based on the given comment.

4. **Comment 4:** Three variables and their scoring methods could be explained in simple words or table for easy clarity.

**Response to comment 4:** Thank you for your comment. The variables and their scoring methods are explained in simple words in the revised manuscript.

5. **Comment 5:** Poor ENCP was also assigned a score of 1. (? Typographical error).

**Response to comment 5:** Thank you very much for identifying this error. It is a typographical error, and corrected.

6. **Comment 6:** There is a mention about 12 independent variables for ENCP. Those could be numbered and clearly mentioned.

**Response to comment 6:** Thank you for your comment. The list of independent variables is removed from the manuscript. Because they are not necessary.

7. **Comment 7:** The scale used to measure wealth index to be mentioned

**Response to comment 7:** Thank you for your comment. Wealth index is well operationalized under the measurement section of the main manuscript.

**Results**

1. **Comment 1:** The description of data mentioned in tables could be avoided

**Response to comment 1:** It is corrected based on the given comment.

2. **Comment 2:** Only 560 of the total 601 study participants were assessed for knowledge on ENCP. The reasons were not mentioned

**Response to comment 2:** Thank you for your comment. First women were asked whether they have an information/awareness about ENCP before asking other knowledge questions. For this question, 41 respondents had no any awareness about ENCP. So, they were not asked for the other knowledge questions.

3. **Comment 3:** Similarly, only 477 were evaluated for knowledge on neonatal danger signs.

**Response to comment 3:** Thank you for your comments. First women were asked whether they have an information/awareness about neonatal danger signs, before asking other knowledge questions. For this question, 124 respondents had no any information/awareness towards neonatal danger signs. So, they are excluded from asking other knowledge questions.

4. **Comment 4:** All the independent variables used and the results of the analysis for each variable could be mentioned.

**Response to comment 4:** Thank you. It is corrected based on the given comment.

5. **Comment 5:** Though used as independent variables, knowledge and attitude on ENCP was not used for analysis.

**Response to comment 5:** Knowledge and attitude on ENCP were assessed along other variables in the bivariable logistic regression, which is used to identify the candidate variables for the final model (Multivariable logistic regression). Unfortunately, the two variables (Knowledge and Attitude had a p-value greater than the cutoff point (0.25), so that, they are not considered as a candidate for the final model. So, they are not included to the final analysis.

6. **Comment 6:** The data collected for reasons for home delivery were not used in analysis. The importance of those data for the study objectives has to be mentioned

**Response to comment 6:** Thank you for your comments. The analysis was done for reasons for home delivery. However, the variable was not a candidate for the final model. This is way it was not used in final analysis.

**Discussion**

1. **Comment 1:** The paragraph mentioning the association of neonatal loss with lack of ENCP is difficult to understand. Reframing sentences for clear understanding is suggested.

**Response to comment 1:** Thank you. The paragraph is reframed.

**Tables**

1. **Comment 1:** Table 2: The frequency of subjects advised on handwashing, drying baby, breastfeeding and those who were not advised do not add to the total of 601 subjects.

**Response to comment 1:** Thank you very much for your comment. The variables that you mentioned are asked for those whose home was visited by HEWs (453).

2. **Comment 2:** Tables 3 & 4: The number of respondents for each category is varying and not all study subject’s data were not studied.

**Response to comment 2:** Thank you very much for identifying this issue. For Table 3, First, the respondents were asked whether they have an information/awareness on ENCP. Then, only those who had an awareness/information (560) were asked the subsequent questions in the table.

For Table 4, first respondents were asked whether they have an information/awareness on neonatal danger signs. Then, only those who had an awareness/information (477) were asked the subsequent questions in the table.

**Once again, thank you very much for your constructive comments!**

**Response to Reviewer 3’s comments**

Dear Reviewer, thank you very much for your valuable and constructive comments that can improve the quality of this paper.

1. **Comment 1:** Authors need to pay attention to the English as some of the sentences need grammatical correction so that they can be meaningful. For example, the section on population in section 2.2 under methods- does not convey any meaning.

**Response to comment 1:** Thank you very much for your valuable comments. The typos and grammatical errors throughout the manuscript, including the section mentioned as an example in the comment are corrected.

**Once again, thank you very much for your constructive comments!**
