# Supplementary material for: Essential newborn care practices and associated factors among home delivered mothers in Guto Gida District, East Wollega zone
Source: PLOS Glob Public Health. 2023 Jan 20;3(1):e0001469. doi: 10.1371/journal.pgph.0001469 (PMC10021559; doi:10.1371/journal.pgph.0001469)
Supplement: Attachment — Submitted filename: Authors Response to reviewers comments.docx [file pgph.0001469.s004.docx]

**Author’s response to reviews**

**Title:** Essential newborn care practices and associated factors among home delivered mothers in Guto Gida District, East Wollega Zone

**Authors:**

Mulugeta Abebe ([mulugetaabebe2012@gmail.com](mailto:mulugetaabebe2012@gmail.com))

Gemechu Kejela ([gemechukejela86@gmail.com](mailto:gemechukejela86@gmail.com))

Melese Chego (melesegershom@gmail.com)

Markos Desalegn ([markosdesalegn@gmail.com)](mailto:markosdesalegn@gmail.com)2)

Version: 2 Date: 30 October 2022

Dear Editor, thank you for providing us an opportunity to revise the paper entitled **" Essential newborn care practices and associated factors among home delivered mothers in Guto Gida District, East Wollega Zone"**. We have revised the paper to address the comments of the reviewers. Below is our point-by-point response to the comments.

Below please find our responses to the editor and three reviewers’ comments.

**Responses to the editor's comments**

Thank you very much for your valuable comments.

1. After incorporating all the comments provided by editor and reviewers, grammatical and typical errors in the manuscript are thoroughly edited.
2. All changes to the manuscript are indicated in the text by highlighting with yellow color.

**Response to the Editor’s comments**

Thank you very much for your valuable comments.

**Comment-1:** Please review your reference list to ensure that it is complete and correct.

**Response to comment-1:** Reference list is reviewed, and all are complete and correct.

Comment 2: Change the figure file to .tiff format and upload separately. In addition, include the supplementary material legend after the reference list.

**Response to comment 2:** The list of figures is changed to .tiff file and uploaded as a separate file. In addition, the number of figures are reduced from 5 to 2. The legend of supplementary information is also placed next to the reference list.

**Response to Reviewer 1’s comment**

**Comment-1:** The manuscript must describe methodologically and ethically rigorous research with conclusions that are appropriately drawn based on the data presented.

**Response to comment-1:** Thank you very much for your valuable comment. The whole body of the manuscript is improved.

**Comment-2:** Spellings of sections headers like “conclusion”, “Introduction”, “Key word” and “study” needs correction. Many needs correction in text, tables and figures.

**Response to comment-2:** Thank you very much for your comment. The spelling of indicated sections corrected and highlighted in the main manuscript. Corrections are also made in text, tables and figures.

**Comment-3:** Methodology: The detailing of “Yes/No and 1/0” can be removed as you have mentioned definitions. Keep categorizations only when authors have more than >2 categories exist.
Sample size formula can be removed.

**Response to comment-3:** Thank you very much for your constructive comment. The detailing of ‘Yes/No and 1/0 are removed. The sample size formula is also removed.

**Comments to Reviewer 2**

**Comment-1:** All comments have been addressed. The language of the manuscript is now easy to understand.

**Response to comment-1:** Thank you very much for your comment.
